# ESTIMATING BASAL ENERGY EXPENDITURE IN LIVER TRANSPLANT RECIPIENTS: THE VALUE OF THE HARRIS-BENEDICT EQUATION

**DOI:** 10.1590/0102-6720201600030013

**Published:** 2016

**Authors:** Andressa S. PINTO, Marcio F. CHEDID, Léa T. GUERRA, Mario R. ÁLVARES-DA-SILVA, Alexandre de ARAÚJO, Luciano S. GUIMARÃES, Ian LEIPNITZ, Aljamir D. CHEDID, Cleber R. P. KRUEL, Tomaz J. M. GREZZANA-FILHO, Cleber D. P. KRUEL

**Affiliations:** 1 Postgraduate Program in Surgical Sciences;; 2 Division of Gastrointestinal Surgery and Liver and Pancreas Transplantation;; 3 Unit of Dietary Therapy;; 4 Division of Gastroenterology and Hepatology;; 5 Division of Medical Statistics, Hospital de Clínicas de Porto Alegre, Federal University of Rio Grande do Sul, Porto Alegre, RS, Brazil

**Keywords:** Liver transplantation, Basal Energy Expenditure, Indirect Calorimetry, Bioelectrical Impedance, Harris-Benedict equation, Mifflin-St, Jeor equation.

## Abstract

**Background:**

Reliable measurement of basal energy expenditure (BEE) in liver transplant (LT) recipients is necessary for adapting energy requirements, improving nutritional status and preventing weight gain. Indirect calorimetry (IC) is the gold standard for measuring BEE. However, BEE may be estimated through alternative methods, including electrical bioimpedance (BI), Harris-Benedict Equation (HBE), and Mifflin-St. Jeor Equation (MSJ) that carry easier applicability and lower cost.

**Aim:**

To determine which of the three alternative methods for BEE estimation (HBE, BI and MSJ) would provide most reliable BEE estimation in LT recipients.

**Methods:**

Prospective cross-sectional study including dyslipidemic LT recipients in follow-up at a 735-bed tertiary referral university hospital. Comparisons of BEE measured through IC to BEE estimated through each of the three alternative methods (HBE, BI and MSJ) were performed using Bland-Altman method and Wilcoxon Rank Sum test.

**Results:**

Forty-five patients were included, aged 58±10 years. BEE measured using IC was 1664±319 kcal for males, and 1409±221 kcal for females. Average difference between BEE measured by IC (1534±300 kcal) and BI (1584±377 kcal) was +50 kcal (p=0.0384). Average difference between the BEE measured using IC (1534±300 kcal) and MSJ (1479.6±375 kcal) was -55 kcal (p=0.16). Average difference between BEE values measured by IC (1534±300 kcal) and HBE (1521±283 kcal) was -13 kcal (p=0.326). Difference between BEE estimated through IC and HBE was less than 100 kcal for 39 of all 43patients.

**Conclusions:**

Among the three alternative methods, HBE was the most reliable for estimating BEE in LT recipients.

## RESUMO

**Racional:**

Estimativa confiável do metabolismo basal em pacientes transplantados de fígado é necessária para adaptar os requerimentos energéticos, melhorar o estado nutricional e prevenir ganho de peso. Calorimetria indireta (CI) é o padrão-ouro para a medição do metabolismo basal. No entanto, ele pode ser estimado utilizando-se métodos alternativos, incluindo a bioimpedância (BI), a Equação de Harris-Benedict (EHB), e também a Equação de Mifflin-St. Jeor (MSJ). Esses métodos alternativos possuem aplicabilidade mais fácil e custo inferior quando comparados à CI.

**Objetivo:**

Determinar qual dos três métodos alternativos para a estimativa do metabolismo basal (EHB, BI e MSJ) seria o mais confiável em pacientes transplantados de fígado.

**Métodos:**

Foi realizado estudo transversal prospectivo incluindo pacientes transplantados de fígado com dislipidemia, em acompanhamento ambulatorial. Comparações dos valores calculados de metabolismo basal via CI aos valores estimados por cada um dos três métodos alternativos (EHB, BI e MSJ) foram realizadas utilizando o de Bland-Altman e o teste de Wilcoxon-Mann-Whitney.

**Resultados:**

Quarenta e cinco pacientes foram incluídos com idade 58±10 anos. O metabolismo basal medido via CI foi 1664±319 kcal para pacientes do gênero masculino, e 1409±221 kcal para o feminino. A diferença média entre a taxa de metabolismo basal aferida por CI (1534±300 kcal) e estimada por BI (1584±377 kcal) foi +50 kcal (p=0.0384). A diferença média entre a taxa de metabolismo basal aferida via CI (1534±300 kcal) e estimada por MSJ (1479.6±375 kcal) foi -55 kcal (p=0.16). A diferença média entre os valores de taxa de metabolismo basal medidos via CI (1534±300 kcal) e estimados por EHB (1521±283 kcal) foi -13 kcal (p=0.326). Além disso, a diferença entre a taxa de metabolismo basal estimada via CI e a aferida por EHB foi menor que 100 kcal para 39 de todos os 43 pacientes avaliados.

**Conclusões:**

A EHB foi o mais confiável dos três métodos de estimativa da taxa de metabolismo basal em pacientes transplantados de fígado em acompanhamento ambulatorial.

## INTRODUCTION

Although hypolipidemia may be a common finding in cirrhotic patients^3^, there is a rising prevalence of obesity and metabolic syndrome after liver transplantation^1^ ^,^ ^11^. Accurate estimation of the basal energy expenditure (BEE), in liver transplant (LT) recipients is necessary to guide improvements on nutritional status and prevent weight gain^2^. 

Indirect calorimetry (IC) is considered as the gold standard method for measuring BEE. However, it has technical limitations that include the need for well trained personnel and an elevated cost^2^. There are other methods for estimating the BEE that are easier to apply and less costly than IC. Among alternative methods for estimating the BEE - also known as Basal Metabolic Rate (BMR) - stand Bioelectrical Impedance (BI), the Harris-Benedict equation (HBE) and Mifflin-St. Jeor equation (MSJ). 

The aim of this study was to measure BEE in LT recipients through IC and compare IC-calculated BEE values to those estimated through three alternative methods BI, HBE and Mifflin-St. Jeor equation (MSJ). 

## METHODS

This is a prospective cross-sectional study that includes all adult LT recipients on dietary outpatient follow-up for dyslipidemia at our service. All patients were selected from a cohort of 199 adult who received a whole-graft LT from a deceased donor at our institution between 2002 and 2014. This study was approved by local IRB and all patients consented before being enrolled.

Inclusion criteria were age >18 years-old, dyslipidemia and at least two months of post-transplant follow-up. Patients who were on drug treatments for dyslipidemia, patients who were using alcohol, handicapped and those who did not consent, were excluded. 

 All patients underwent evaluation by a dietician before being enrolled. Body weight, height, body mass index (BMI) and waist were measured. BEE was calculated using IC, and also estimated through BI, HBE and Mifflin-St. Jeor Equation (MSJ) by a single researcher (Pinto, A. S.). 

###  Indirect calorimetry (IC)

IC is a noninvasive method for calculating BEE by utilizing the volume of expired oxygen and production of carbon dyoxide obtained through analyzing the air expired by the lungs^3^. BEE was estimated in a thermoneutral environment (Metabolic Gas Analyzer VO 2000, Software Aerograph Breeze, Medical Graphics - Cardiorespiratory Diagnostic Systems), after the patient was fasting for at least 6 h. Patients rested for at least 30 min before data were collected. The system was adjusted before each measurement. Oxygen consumption and CO2 production were measured after the patient stood in supine position for at least 25 min. 

### Bioelectrical impedance (BI)

Patients were instructed to fast for at least 8 h before the exam, and not to practice physical activities during 24 h preceding the exam. BMI analyzer (model Bodystat(r) 1500) utilizes four small probes, being one applied on right hand and another on right wrist, a third on right ankle and the last one on right foot. BI measures were performed on the right side of the body. Patient was positioned on supine position, with both legs in contact and arms not touching the body^12^. Lean mass and fat mass were measured through BI.

### Harris-Benedict Equation (HBE)

For estimating BEE through using the Harris-Benedict Equation (HBE) (kcal/day) the following equation was utilized for male gender: 66.47 + (13.75 x weight in kg) + (5.003 x height in cm) - (6.775 x age in years). For female gender, the following equation was utilized: 655.09 + (9.563 x weight in kg) + (1.85 x height in cm) - (4.676 x age in years)^4^.

### Mifflin-St. Jeor Equation (MSJ)

For estimating BEE through using the MSJ (kcal/day) the following equation was utilized for male gender: 10 x weight (kg) + 6.25 x height (cm) - 5 x age (y) + 5. For female gender, the following equation was utilized: 10 x weight (kg) + 6.25 x height (cm) - 5 x age (y) - 161^14^.

### Statistical analysis

Categorical variable comparisons were performed utilizing Chi-Square test. Regarding the high cost involved with utilizing indirect calorimetry and patient visits, BEE measurements were performed only once. Numeric variables were compared utilizing T test and/or Mann-Whitney U test as appropriate. Univariate analysis performed through simple linear regression analyzed association of demographic variables (percentage of lean mass, age and BMI), to BEE calculated through IC. "X" variable was each demographic variable, and BEE was the "Y" variable. The association of gender ("X" variable) and BEE ("Y" variable) was analyzed through one-way Wilcoxon Rank Sum Test. Variables exhibiting p value <0.1 were selected for multivariate analysis using multiple linear regressions. Bland-Altman method was utilized for comparisons between the three alternative methods for estimating BEE, BI, HBE and MSJ to the gold standard IC. JMP Statistical Discovery version 12 (SAS, Cary, NC, USA) and was utilized for statistical analyses. Excel for Windows (Redmond, Washington, US) was utilized for construction of Bland-Altman plots. p-values <0.05 were considered as statistically significant. 

## RESULTS

Forty-five patients in post-transplant outpatient follow-up (two months to 11 years post-transplant) were included in this study. These were 22 male and 23 female patients, mean age 58±10 years (Tables 1 and 2).

**TABLE 1:** Demographic and anthropometric characters for the whole study cohort (n=45).

Variable	Value (%)
Gender - n (%)	
Male	22 (48,88%)
Female	23 (51,11%)
Age (years) - Mean±Std. Dev.	58 ± 10
BMI (kg/m²) - Mean ± Std. Dev.	27.83 ± 5.38
CW (cm) - Mean ± Std. Dev.	94.3 ± 13.80
LM (%) - Mean ± Std. Dev.	66.14 ± 7.56
FM (%) - Mean ± Std. Dev.	33.81 ± 7.53

BMI=body mass index; CW=circumference waist; LM (%)=lean mass; FM(%)=fat mass

**TABLE 2:** Demographic and anthropometric characters stratified by gender

Male (n=22)	Female (n=23)
BMI=27.92 ± 5.43	BMI=27.74 ± 5.45
Neck circumference=99.75±13.5	Neck circumference=89.1±12.2
%Lean mass=70.5±5.1	% Lean mass=61.9±7.2
%Fat mass=29.5±5.1	% Fat mass=38±7.2

BMI=body mass index

Twenty five patients were aged less than 60 years-old, and 20 aged 60 or older. Patients younger than 60 years had a mean BMI of 28.6±5.9, the mean BMI being 26.9±4.6 for older than 60 years. All 45 patients had undergone LT more than six months prior to being evaluated in this study. Thirty-eight of the total 45 patients were evaluated after standing at least one year after LT. 

**FIGURE 1:**
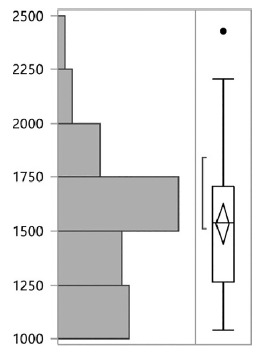
BEE distribution for the whole sample using IC

Mean IC-calculated BEE was 1534±300 (Figure 1). Mean BEE as estimated through IC was 1664±319 kcal for male and 1409±221 kcal for female patients (p=0.004). For the entire cohort, percentage of mean lean mass was 66.14%±7.6. For the entire cohort, univariate analysis (simple linear regression) revealed age not to be associated to an increase or reduction in the BEE (p=0.2). Percentage of lean mass was not associated to difference in the BEE (p=0.78). An increase in the BMI was associated to an increase in the BEE (p=0.0001). Multivariate analysis utilizing the two factors that were associated to an increase in the BEE (male gender and BMI) revealed both male gender (p=0.0001) and BMI (p=0.0001) as independently associated to an increase in the BEE.

**FIGURE 2:**
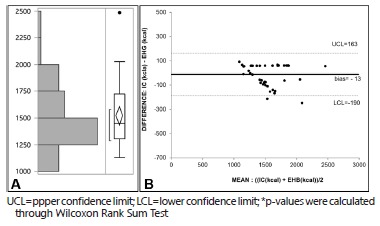
A) BEE distribution for the whole sample using HBE; B) Bland-Altman Plot comparing BEE calculated through IC and HBE, (p=0.326)*

Mean HBE-calculated BEE was 1521±283 (Figure 2A). As estimated using the Bland-Altman method, bias of BEE measured through IC (1534±300) and estimated through HBE was -13 kcal. Upper confidence limit (UCL) was 163 kcal, and lower confidence limit (LCL) was -190 kcal (confidence interval=353 kcal, Figure 2B). As estimated through Wilcoxon Rank Sum Test, the difference between BEE calculated through IC and estimated through HBE was not statistically significant (p=0.326). This difference was higher than 10% for only two patients. Also, this difference was higher than 100 kcal for only six out of 45 total patients. 

**FIGURE 3:**
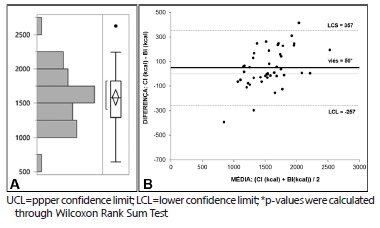
A) BEE distribution for the whole sample using BI; B) Bland-Altman Plot comparing BEE calculated through IC and BI (p=0.038)*

Mean BI-calculated BEE was 1584±377 (Figure 3A). As estimated using the Bland-Altman method, bias of BEE measured through IC (1534±300) and estimated through BI was +50 kcal. Upper confidence limit (UCL) was 357 kcal, and lower confidence limit (LCL) was -257 kcal (confidence interval=500 kcal) (Figure 3B). As estimated through Wilcoxon Rank Sum Test, the difference between BEE calculated through IC and estimated through BI was statistically significant (p=0.038). This difference was higher than 10% for 13 of the total 45 patients. Also, this difference was higher than 100 kcal for 19 out of 45 patients.

**FIGURE 4:**
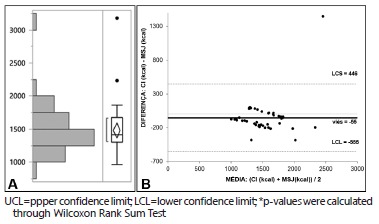
A) BEE distribution for the whole sample using MSJ; B) Bland-Altman Plot comparing BEE calculated through IC and MSJ (p=0.16)*

Mean MSJ-calculated BEE was 1480±375 (Figure 4A). As estimated using the Bland-Altman method, bias of BEE measured through IC (1534±300) and estimated through MSJ was -55 kcal. Upper confidence limit (UCL) was 446 kcal, and lower confidence limit (LCL) was -555 kcal (confidence interval=1,001 kcal) (Figure 4B). As estimated through Wilcoxon Rank Sum Test, the difference between BEE calculated through IC and estimated through BI was not statistically significant (p=0.16). This difference was higher than 10% for 13 patients. Also, this difference was higher than 100 kcal for 21 out of the total 45 patients. 

## DISCUSSION

A recent study revealed an overweight prevalence of 45% for LT recipients by the end of the first post-transplant year^1^. During the second this prevalence was 60%, being as high as 70% on the third post-transplant year^16^ ^,^ ^18^. The present study revealed an overweight prevalence that is similar to previous studies^17^ (55% for patients aged less than 60 years and 30% for patients aged older than 60 years-old). 

In the present study, mean BEE calculated through using IC was 1534 kcal. BEE was statistically higher for male patients than for females. As to age, an increased age was associated to a decreased BEE^21^. The only two independent predictors for an increased in the BEE estimated through IC were male gender and BMI. Thus, neither age nor percentage of lean mass were associated to an increased BEE. Richardson *et al.* followed 23 patients until they reached the 9^th^ post-transplant month, and observed that a lower BEE as calculated through IC was an important predictor for fat mass gain after liver transplant^6^. 

Besides the gold standard IC, there are alternative methods for estimating BEE such as BI, HBE and MSJ. Clinical use of HBE has been debated^5^ ^,^ ^8^ ^-^ ^9^ ^,^ ^13^ ^,^ ^15^ ^,^ ^19^ ^,^ ^22^. A study analyzing healthy subjects aged 18-30 years, using the HBE underestimated the BEE in 2.91% for female and 6.61% for male (p<0.05)^4^. Two other studies, the first analyzing patients undergoing elective surgery and the other analyzing patients suffering from liver failure detected significant differences between energy consumption measured through IC and the one estimated through HBE. In one study, HBE underestimated the energetic needs by 25%^10^. Another study from our institution detected HBE to overestimate BEE as compared to IC^17^. 

This is the first study comparing BEE estimated through alternative methods (HBE, BI and MSJ) to the one measured through IC in LT recipients. In the present research, HBE underestimated BEE. However, the mean difference was small (13 kcal), not statistically significant, and with a much lower confidence interval as compared to the other two alternative methods (BI and MSJ). This suggests that HBE is more reliable for determining BEE in LT recipients in outpatient follow-up than the other two alternative methods (BI and MSJ). Moreover, the difference of BEE calculated through IC and estimated through HBE was higher than 100 kcal for only six out of 45 total patients, being higher than 10% for only two patients. Thus, in the present study sample, HBE proved as the most reliable alternative method for estimating BEE. 

The limitations of this study pertain to its inclusion criteria (dyslipidemic liver recipients), which could have tended towards selecting the most obese of our LT recipients. However, considering that overweight prevalence was similar to that of LT recipient populations analyzed in prior studies, it is likely that the findings from this study can be generalized to the LT recipient pool^1^ ^,^ ^16^ ^-^ ^17^.

Weight control measures are warranted to control weight gain in LT recipients and prevent obesity in this patient population. Male LT recipients have a BEE that is statistically higher than female patients who have a similar BMI. For individuals of the same gender, an increased BMI is associated to an increased BEE. HBE seems to be a reliable method for estimating the BEE in LT recipients on outpatient follow-up. Whenever there is unavailability of IC, HBE rather than BI or MSJ should be the method of choice for estimating the BEE in LT recipients.

## CONCLUSION

Among the three alternative methods (HBE, BI and MSJ), HBE was the most reliable for estimating BEE in LT recipients.
